# Enhancement of the Stability and Anti-DPPIV Activity of Hempseed Hydrolysates Through Self-Assembling Peptide-Based Hydrogels

**DOI:** 10.3389/fchem.2018.00670

**Published:** 2019-01-24

**Authors:** Carmen Lammi, Carlotta Bollati, Fabrizio Gelain, Anna Arnoldi, Raffaele Pugliese

**Affiliations:** ^1^Department of Pharmaceutical Sciences, University of Milan, >Milan, Italy; ^2^Tissue Engineering Unit, Institute for Stem Cell Biology, Regenerative Medicine and Innovative Therapies-ISBReMIT, Fondazione IRCSS Casa Sollievo della Sofferenza, >San Giovanni Rotondo, Italy; ^3^Center for Nanomedicine and Tissue Engineering, ASST Grande Ospedale Metropolitano Niguarda, Milan, Italy

**Keywords:** bioactive peptides, DPPIV, nano-nutraceutical, rheology, self-assembling peptide

## Abstract

Although there is an increasing interest for bioactive food protein hydrolysates as valuable ingredients for functional food and dietary supplement formulations, their potential applications are hampered by their insufficient stability in physiological conditions. In this study, an innovative strategy based on nanomaterials was developed in order to increase the hempseed hydrolysate stability and the anti-diabetic properties, through their encapsulation into ionic self-complementary RADA16 peptide based-hydrogels. Atomic force microscope (AFM) morphological analysis indicated that the new nanomaterials were composed of a nanofibril network, whose increased diameter in respect to native RADA16 suggests the presence of transient non-covalent interactions among the RADA16 supramolecular assemblies and the embedded hempseed peptides. Structural analysis by FT-IR spectroscopy indicated typical β-sheet signatures. The RADA16-hempseed protein hydrolysate hydrogel was shown to act as a novel dipeptidyl peptidase IV (DPPIV) inhibitor in different biological assays. Finally, this nanoformulation was used as a drug delivery system of the anti-diabetic drug sitagliptin, helping to reduce its dosage and eventually associated side-effects.

## Introduction

Bioactive peptides are increasingly recognized as valuable ingredients for the formulation of functional foods and dietary supplements providing useful health benefits (Arnoldi et al., 2015). They are rarely present in foods as such, whereas in most cases they are encrypted in protein sequences and may be delivered by digestion, enzymatic hydrolysis, or fermentation (Nongonierma et al., 2017). Different biological activities are currently under investigation, in particular, the hypotensive (Girgih et al., 2014a), hypocholesterolemic (Arnoldi et al., 2015), and hypoglycemic ones (Lammi et al., 2016b).

Protein hydrolysates may be obtained either from animal or plant proteins: in this context, hempseed proteins may represent an innovative source of bioactive peptides, since this seed contains more than 25% protein that can be hydrolyzed in different conditions using either digestive or food grade enzymes (Aiello et al., 2016). Enzyme/substrate ratios, pH, time, and temperature influence in a significant way the peptides release with a direct effect on bioactivity (Aiello et al., 2017; Zanoni et al., 2017). Only a few research groups are actively pursuing the biological activity of hydrolyzed hempseed proteins with specific interests for the antioxidant (Girgih et al., 2011, 2013, 2014b), hypocholesterolemic (Zanoni et al., 2017), and hypotensive activities (Girgih et al., 2014a; Malomo et al., 2015), whereas other biologically properties, such as the anti-diabetic one, have been rarely considered (Nongonierma and Fitzgerald, 2015a).

In this context, dipeptidyl peptidase IV (DPPIV)/CD26 is considered an interesting anti-diabetic target. This ectoenzyme (EC 3.4.14.5), ubiquitously expressed on the surface of various cell types, such as intestinal epithelial cells (Abbott et al., 1994), cleaves dipeptides from the N-terminus of polypeptides in which proline is at the penultimate position. A truncated soluble form of this enzyme, which possesses a similar activity, is also found in serum. Enhanced serum DPPIV levels and/or activity have been suggested to be correlated with many metabolic diseases, such as type 2 diabetes (T2DM), obesity, cardiovascular disease, and non-alcoholic fatty liver disease (NAFLD) (Röhrborn et al., 2015; Nargis and Chakrabarti, 2018). This is considered a promising therapeutic target for glycemic control, because it plays a key role in glucose metabolism by N-terminal truncation and inactivation of the incretins glucagon-like peptide (GLP)-1 and gastrointestinal insulinotropic peptide (GIP) that together are responsible for up to 70% of post-prandial insulin secretion (Nauck et al., 2004). Since these hormones are rapidly inactivated by DPPIV (Doupis and Veves, 2008), the inhibition of this enzyme promotes insulin secretion and reduces glucagon release.

Even though synthetic DPPIV inhibitors are currently available on the market, several food-derived peptides and hydrolysates have been identified and found to act as promising DPPIV inhibitors (Lammi et al., 2016b; Nongonierma et al., 2018). Having verified that this kind of information was not available for hempseed peptides, this study's first aim was to evaluate the DPPIV inhibitory activities exerted by hempseed protein hydrolysates obtained by treating hempseed proteins with trypsin (HT) or pepsin (HP). The activity was assessed by different complementary methods, in particular an *in vitro* commercial test based on the purified enzyme, an *in situ* assay on Caco-2 cells, and an *ex vivo* test on human serum samples.

Although there are experimental evidences that some food-derived peptides may be absorbed at least in part at intestinal level (Lammi et al., 2016a), their low stability and bioavailability still remain major concerns for practical applications. These issues, however, may be overcome by the use of well-designed and controlled delivery systems (Park, 2014; Lopalco and Denora, 2018). The development of an efficient, biocompatible, and bio-absorbable release system requires the use of materials capable of delivering the bioactive compounds in a controlled manner, such as self-assembling peptide-based hydrogels (SAPs). SAPs are recognized as useful tools in a broad range of biomedical and biotechnological applications, ranging from carriers for drug (Koutsopoulos et al., 2009; Gelain et al., 2010) or pesticides delivery (Bolat et al., 2018), scaffolds for regenerative medicine (Pugliese and Gelain, 2017), to actuators for optics and fluidics (Tao et al., 2017). Usually, SAPs are short peptides (8–16 residues) containing alternate charged hydrophilic and hydrophobic amino acids that, upon exposure to physiological conditions of pH, temperature, or electrolytes, spontaneously self-organize into interwoven nanofibers with diameters of 10–20 nm (Pugliese and Gelain, 2017). Peptide hydrogels are easy to use, biodegradable, non-toxic, non-immunogenic, and non-thrombogenic. They are generally more biocompatible (Zhang, 2003) than polymeric materials, such as PLLA, PLGA, PCL, and their degradation products are easily metabolized into natural amino acids. Moreover, molecular design permits to tailor SAPs sequences for specific application needs.

Taking all this information into account, the second objective of the study was to verify whether it was possible to enhance the stability of hempseed protein hydrolysates by combining them with the ionic self-complementary peptide RADA16 (i.e., Ac-RADARADARADARADA-CONH2), a well-known and characterized SAP based-hydrogel (Zhang et al., 1993). This objective was achieved by performing different experiments in order to demonstrate the feasibility of this encapsulation procedure without disturbing the overall stability of the RADA16 cross-β secondary structures, to assess the morphology and biomechanics of the obtained nanofibers, and to evaluate the biological activity of these materials on DPPIV.

Finally, since previous literature has shown that interesting synergistic effects may be observed when milk peptides are combined with sitagliptin (Nongonierma and Fitzgerald, 2015b), one of the main DPPIV inhibitors successfully commercialized as oral drug for the treatment of T2DM, the third objective of the study was aimed at evaluating the possible synergistic activity of the RADA16-hemp hydrogels and sitagliptin.

## Results and Discussion

### Evaluation of the Inhibitory Activity of HT and HP Hydrolysates on DPPIV

**Figure 1:**
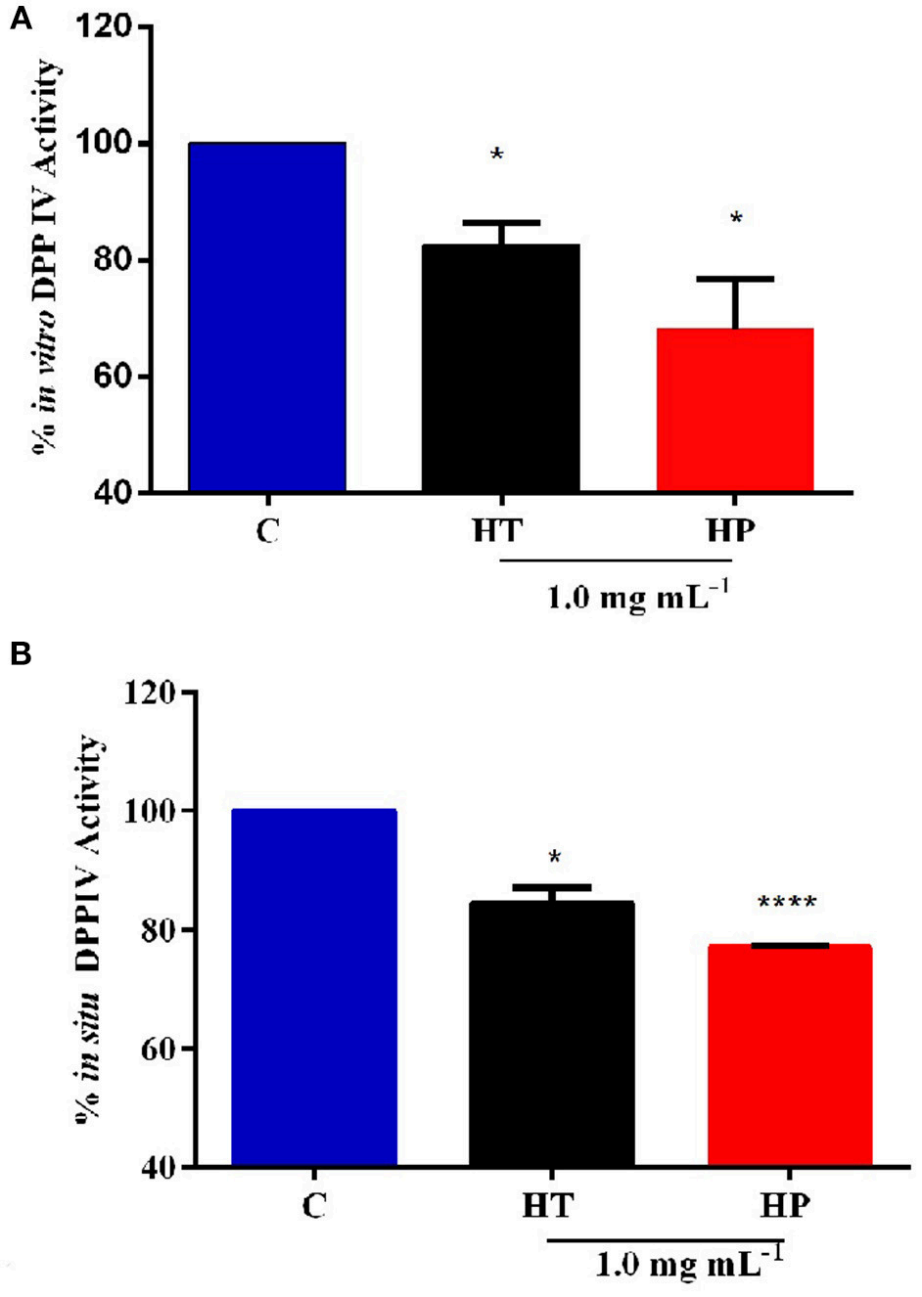
Inhibition of DPPIV activity by HT and HP hydrolysates evaluated with different methods. **(A)** HT and HP hydrolysates (1.0 mg mL^−1^) inhibit *in vitro* the activity of human recombinant DPPIV by 17.5 ± 2.7 and 32.0 ± 6.2%, respectively. **(B)** HT and HP hydrolysates (1.0 mg mL^−1^) inhibit *in situ* the DPPIV activity in non-differentiated human Caco-2 cells by 15.5 ± 1.8 and by 22.5%, respectively. *p > 0.05; ****p > 0.0001.

In order to assess the anti-diabetic properties of HT and HP hempseed protein hydrolysates, their ability to decrease *in vitro* the DPPIV activity was evaluated as a first biochemical approach. Preliminarily, HT and HP hydrolysates were tested *in vitro* at 0.5 and 1.0 mg mL^−1^, respectively. Results suggest that HP is more active at 1.0 mg mL^−1^, whereas HT shows comparable inhibitory activities at both concentrations (Figure S1). For this reason, it was decided to carry out all further experiments at 1.0 mg mL^−1^ as the best concentration for both hydrolysates. Figure 1A clearly indicates that the HT and HP hydrolysates at 1.0 mg mL^−1^ inhibit *in vitro* DPPIV activity by 17.5 ± 2.7% and 32.0 ± 6.2%, respectively. The fact that the HP hydrolysate is 2-fold more active than the HT one highlights the importance of the specificity of the enzyme used to release the peptides from the parent proteins. The bioactivity of a single peptide is typically related to its size and amino acid sequence, whereas that of a protein hydrolysate depends strictly on its total composition, including inactive and active species and possible synergistic or antagonist effects (Aiello et al., 2017; Zanoni et al., 2017).

The inhibitory activity of these hydrolysates on DPPIV is in agreement with those of other food protein hydrolysates (Lacroix and Li-Chan, 2012). For instance, simulated gastrointestinal digestion of the intact rice and hemp proteins yielded DPPIV IC_50_ values between 1.85 ± 0.34 and 4.50 ± 0.55 mg mL^−1^, respectively (Nongonierma and Fitzgerald, 2015a). Moreover, a hydrolysate of Atlantic salmon skin gelatin generated using Flavorzyme® inhibits the DPPIV activity by 45.0% at 5.0 mg mL^−1^ (Li-Chan et al., 2012), whereas a hydrolysate prepared by hydrolyzing Japanese rice bran using Umanizyme G® inhibits the DPPIV activity with an IC_50_ value equal to 2.3 mg mL^−1^ (Hatanaka et al., 2012). It is important to underline that all these studies have been performed only using an *in vitro* tool in which porcine DPPIV is involved. Although the sequence is highly conserved among mammalian species, human, and porcine DPPIV enzymes have only an 88% sequence identity and there are evidences that porcine and human DPPIV differ in their susceptibility to inhibition by food-derived peptides (Lacroix and Li-Chan, 2015). Since the inhibition is stronger on the porcine DPPIV enzyme than on the human one, the usage of the former to assess the inhibitory effect may lead to an overestimation of the potency or effectiveness on human DPPIV (Lacroix and Li-Chan, 2015). This aspect clearly underlines the need to deeply investigate the DPPIV inhibitory effects of food-derived peptides not only from a biochemical point of view but also at human cellular level (Lammi et al., 2018).

Further experiments were therefore performed using a cellular assay based on non-differentiated human intestinal Caco-2 cells, which has been recently developed and optimized by us as a useful tool for the screening and identification of new DPPIV inhibitors (Lammi et al., 2018). These cells express several morphological and functional characteristics of enterocytes, possess a wide range of membrane peptidases naturally expressed by the apical side of enterocytes, DPPIV included, and are useful to investigate the potential metabolism of tested compounds. Caco-2 cells were treated with 1.0 mg mL^−1^ of HT and HP hydrolysates for 24 h, then the AMC-Gly-Pro substrate (50.0 μM) was added, and the fluorescence signals were detected by a plate reader. Figure 1B shows that the HT hydrolysate inhibited the DPPIV activity by 15.5 ± 1.8% and HP hydrolysate by 22.5 ± 0.19%. These results confirm the *in vitro* tests although they also indicate that the incubation with the Caco-2 cells slightly impairs the inhibitory potencies of the hydrolysates, with a greater effect on the HP one. This may be possibly explained by metabolic effects.

A very critical issue in the practical application of food peptides regards the low stability especially in respect to the proteases present in the biological samples, in particular in serum. Therefore, *ex vivo* experiments were also performed spiking human serum samples with HT and HP hydrolysates at the concentration of 1.0 mg mL^−1^ and incubating at 37°C for 24 h. The next day, the AMC-Gly-Pro substrate (50.0 μM) was added and the fluorescence signals were measured. Small inhibitory activities were observed in both cases that were not significant (data not shown), whereas sitagliptin, used as a positive control, reduced the circulating enzyme activity by 68.5 ± 5.3% vs. the control sample. These results are indicative of an extensive degradation of the hydrolysates in this complex environment.

This whole body of information prompted us to develop a new strategy that might overcome the observed limitations and improve the DPPIV inhibitory activity by incorporating them within the nanofibrous structures of RADA16 SAP-hydrogel.

### Self-Assembly of RADA16-HT and RADA16-HP Hydrolysates Into Fibrillar Nanostructures

**Figure 2:**
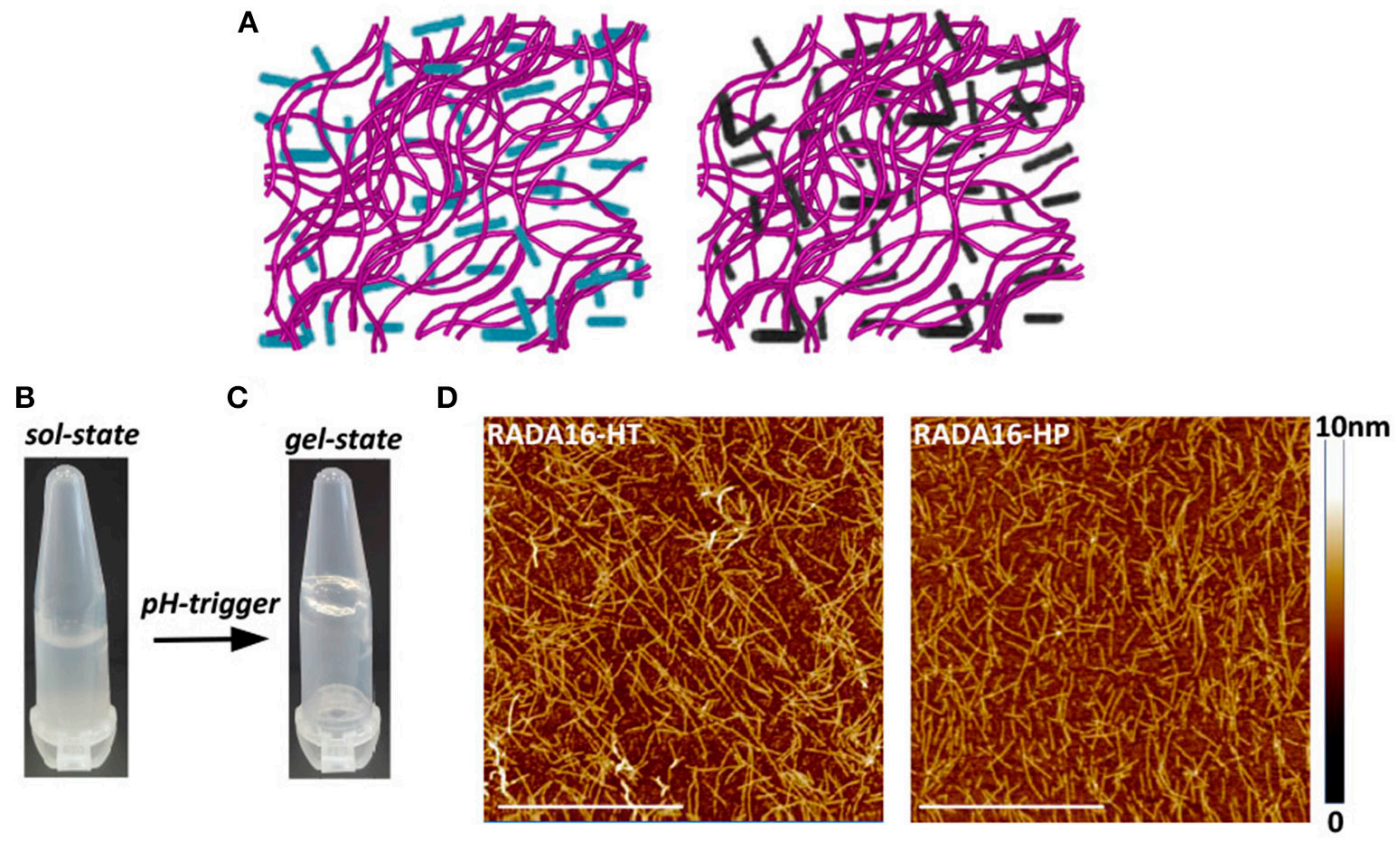
Self-assembly of RADA16-hempseed hydrolysates. **(A)** Cartoon models of RADA16-HT (left) and RADA16-HP (right) peptide based-hydrogels. RADA16 network of nanofibers is shown in purple, while HT and HP are in cyan and dark gray, respectively. **(B)** Photographs of freshly prepared RADA16–hempseed solution and **(C)** after hydrogelation (in 0.09% saline). **(D)** AFM morphological analysis of RADA16-HT and RADA16-HP hydrogels. Images show a network of nanofibrils with ~24 nm diameter and up to 2 nm length (Scale bar, 1 μm).

The ionic self-complementary RADA16 peptide is known to have a strong propensity to spontaneously self-assemble into ordered nanofibrous structures upon exposure to external stimuli (e.g., pH, temperature, monovalent, or divalent electrolyte ions). Typically, RADA16 fibrils are linear with a 10–20 nm diameter and a 2.5–5 nm length (Zhang et al., 1993). At the macroscale, these nanofibers further organize to form highly hydrated hydrogels containing up to 99.5% (w/v) water, which can be easily and harmlessly used as drug delivery systems (Koutsopoulos et al., 2009; Gelain et al., 2010). To assess whether the hempseed hydrolysates modify the self-assembly propensity of RADA16, this material was dissolved in distilled water at the concentration of 10 mg mL^−1^ and mixed with the HT and HP hydrolysates (1.0 mg mL^−1^) at a 3:1 (v/v) ratio. In order to further promote self-assembly, an isotonic saline solution (ionic strength 0.09%) was slowly added to the mixed peptide solutions, and the hydrogels were formed at room temperature. The 3:1 ratio between RADA16 and hempseed peptides was employed, since the increase of RADA16 concentration resulted in a higher density network of nanofibers that may possibly hinder the release of the hempseed peptides, and probably increase the interactions of RADA16 nanofiber-hemp diffusant yielding a decrease in apparent diffusivity (Figure 2A).

The freshly prepared RADA16-hemp hydrolysate solutions were viscous-liquid, clear, and homogeneous (Figure 2B); after the hydrogelation, the solutions took on a gel-like consistency and no noticeable aggregates were visualized (Figure 2C). Atomic force microscope (AFM) morphological analysis, carried out to monitor the effects of HT and HP peptides on the self-aggregated nanostructures of RADA16, showed a network of nanofibrils with ~24 nm diameter and up to 2 nm length (Figure 2D), similar to those previously reported (Yokoi et al., 2005). The slight increase of RADA16-hempseed peptides diameters from AFM imaging, compared to native RADA16, suggests the presence of transient non-covalent interactions (i.e., electrostatic forces, VDW, hydrogen bonds) among the RADA16 supramolecular assemblies and the embedded hempseed peptides (see Table S1 in the ESM).

Overall, these AFM results confirmed the assembly propensity of RADA16-hempseed peptides into nanofibers, highlighting that HP and HT hydrolysates minimally perturb the RADA16 structures, and therefore they can be easily trapped inside the entangled nanofibrous domains of the RADA16 hydrogel, which allows less free motion of the hemp diffusants and facilitate their slow and sustained release.

### Influence of HT and HP Hydrolysates on Assembled Secondary Structures

**Figure 3:**
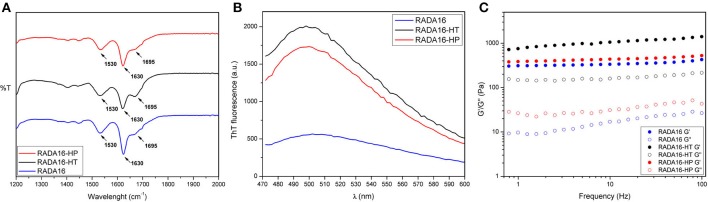
Structural and biomechanical properties of RADA16-HT and RADA16-HP hydrogels. **(A)** ATR-FTIR spectra of RADA16-HT (dark) and RADA16-HP (red) hydrogels closely resemble that of pure RADA16 (blue), displaying typical β-sheet signature in Amide I (1,600–1,700 cm^−1^) and Amide II (1,480–1,575 cm^−1^) regions. **(B)** ThT emission spectra. Pure RADA16 (blue) showed an affinity for ThT ascribable to the presence of cross-β fibril structures, while RADA16-HT (dark) and RADA16-HP (red) produced an increase of ThT fluorescence intensity due to transient electrostatic interactions between the peptides and RADA16 molecules during self-assembly and nanofiber formation. **(C)** Rheological characterization. Assembled hydrogels were monitored by frequency sweep tests (0.1–100 Hz). All tested materials showed typical hydrogel-like profiles featuring a predominant elastic solid-like behavior (G'), as compared with the viscous component (G”). RADA16-HT and RADA16-HP showed a slight increase in the G' values (~750 and ~450 Pa, respectively) along the tested frequency range compared to pure RADA16 (~353 Pa), but they still showed strength profiles typical of soft peptide scaffolds.

Structural analysis of assembled RADA16-hempseed hydrolysates was pursued by attenuated total reflection (ATR) Fourier transform infrared (FT-IR) spectroscopy. As expected, by analyzing the Amide I region (1,600–1,700 cm^−1^), which is mainly associated with C = O stretching vibration and related to the SAP-backbone conformation, native RADA16 showed β-sheet features characterized by the presence of the two components at 1,630 and 1,695 cm^−1^ (Figure 3A). FT-IR spectra of the RADA16-HP hydrolysate and RADA16-HT hydrolysate closely resembled that of the RADA16 in the native state, displaying typical β-sheet signatures. Moreover, in the Amide II region (1,480–1,575 cm^−1^), β-sheet aggregation for all tested RADA16-hempseed hydrolysates was confirmed by the presence of peaks at 1,530 cm^−1^ (directly related to CN stretching and NH bending). Altogether, FT-IR analysis confirmed self-aggregation of tested RADA16-hempseed hydrolysates into β-sheets, suggesting that the introduction of hempseed hydrolysates did not affect the macromolecular organization of the RADA16 hydrogel.

Furthermore, to get more information on the RADA16-hempseed hydrolysates capacity of forming cross-β fibril structure, thioflavin T (ThT) spectroscopy assay was carried out. It is widely accepted that β-rich structures feature ThT-binding sites, and that these interactions may give insights on cross-β structures and fibril formation (Biancalana and Koide, 2010). ThT has a weak fluorescence in aqueous environment, with excitation and emission bands centered at ~350 and 440 nm, respectively. Upon binding to β-rich fibrils, bathochromic shifts of both excitation and emission at 440 and 490 nm, respectively, are observed. The emission intensity at ~490 nm is assumed to be directly proportional to the quantity of cross-β fibrils present in the sample. When the probe ThT was applied to RADA16, a characteristic fluorescence emission at ~490 nm confirmed that the peptide had adopted a cross-β-sheet conformation (Figure 3B). Instead, an increase of ThT fluorescence intensity was found when RADA16 was mixed with either HT or HP peptides (Figure 3B). This suggested that hempseed peptides might increase the overall presence of cross-β structures in self-assembled RADA16 hydrogels, probably due to transient electrostatic repulsions that can be formed between hempseed peptides and RADA16 molecules during self-assembly and nanofiber formation. This shows that electrostatic interactions could play a pivotal role in the stability of fibrillar systems as carriers for drug delivery. A reasonable explanation concerning this behavior has been proposed by Zhang et al. (1993) using RADA16 hydrogel as a platform to release lysozyme, trypsin inhibitor, BSA, and IgG (Koutsopoulos et al., 2009). They speculated that SAPs charge may be an important factor affecting not only the fibers stability, but also the interactions and release kinetics when the release occurs through peptide hydrogels consisting of nanofibers that carry a net (positive or negative) charge.

### Biomechanical Properties of RADA16-HT and RADA16-HP Hydrogels

In addition to morphological and structural properties, the biomechanics of the nanostructures play a significant role in translation for applications (e.g., nano-carriers, nano-devices, actuators for optics, and fluidics etc.) (Pugliese et al., 2018a,b). In the case of hydrogels, the most relevant biomechanical features to be characterized are the storage (G′) and loss (G′′) moduli. The former reflects the stiffness trend of the biomaterial, while the latter represents the energy dissipated during the test and correlates with the liquid-like response of the hydrogel. The ratio between G′ and G′′ provides insights of the viscoelastic profile of tested material, i.e., whether it behaves as a viscous liquid (G′ < G′′) or as an elastic solid (G′>G′′). Accordingly, it was crucial to assess the elastic and viscous response of materials, by varying frequencies of applied oscillatory stress (see section Methods for further details), in order to investigate how the HT and HP hydrolysates could influence the mechanical strength of the RADA16 hydrogel. Upon comparing the rheological properties of native RADA16, and RADA16-HT or RADA16-HP hydrolysates (Figure 3C), trends of G′ and G′′ for all species showed typical hydrogel-like profiles, featuring a predominant elastic solid-like behavior (G′), as compared with the viscous component (G′′). The G′ and G′′ values remained relatively constant throughout the test; hence all scaffolds were very resistant to deformation. Nevertheless, RADA16-HP hydrolysates and RADA16-HT hydrolysates showed a slight increase in the elastic shear modulus G′ values (~450 and ~750 Pa, respectively) along the tested frequency range (0.1–100 Hz) at low strains (1%) compared to the native RADA16 (~353 Pa), although they still were showing strength profiles typical of soft peptide scaffolds. This slight increase in the mechanical properties may be attributed to electrostatic interactions taking place between hempseed peptides and RADA16 molecules during self-assembly phenomena that increase the overall presence of β-structures (as investigated in the previous section). Indeed, it is widely accepted that in β-sheet-rich SAPs after self-assembly, interactions among self-assembled fibers lead to increase of G′ values.

Overall, rheological assays showed that the RADA16 hydrogel mixed with the hempseed hydrolysates had stable mechanical features. Also, encapsulation of the hempseed hydrolysates did not alter the RADA16 self-assembling propensity, displaying instead a slight improvement in elastic shear modulus G′. Therefore, on the whole, these results provided insight on the feasibility of hempseed peptides encapsulation that may turn in their smart delivery and sustained release from the nanoformulation, which is an open challenging issue of nanotechnology in the food and agriculture sectors.

### Enhanced DPPIV Inhibitory Activity and Stability of RADA16-HT and RADA16-HP Hydrogels

**Figure 4:**
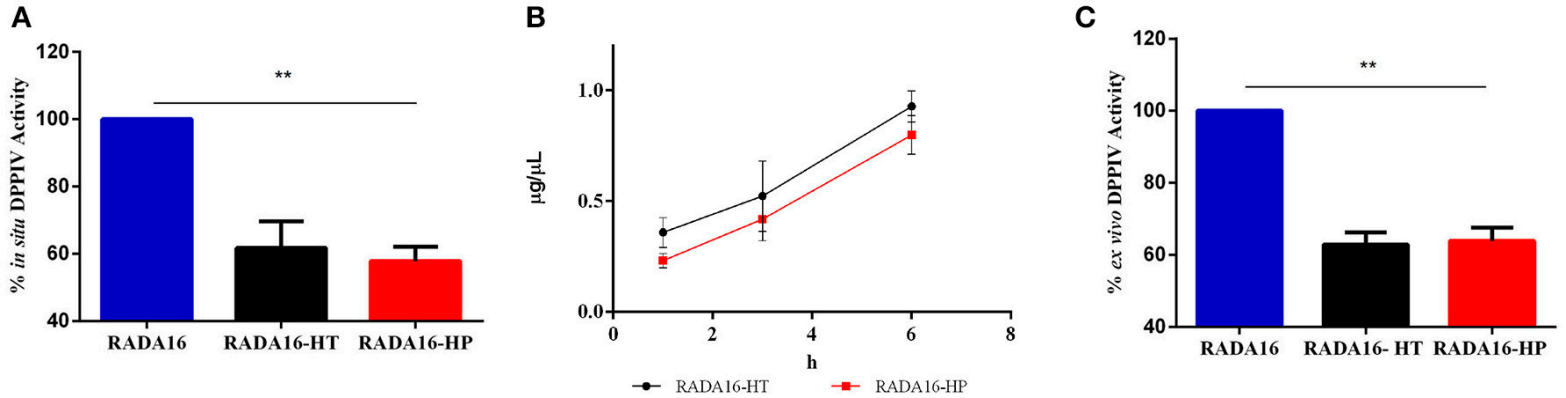
DPPIV inhibitory activity, stability, and release of RADA16-HT and RADA16-HP hydrogels. **(A)** RADA16-HT and RADA16-HP hydrogels (1.0 mg mL^−1^) reduce *in situ* the DPPIV activity in non-differentiated human Caco-2 cells by 38.3 ± 5.6 and 42.2 ± 3.0%, respectively, vs. the RADA16 hydrogel. **(B)** Release of HT and HP peptides from the RADA16 hydrogel as a function of the time. **(C)** RADA16-HT and RADA16-HP hydrogels decrease the circulating DPPIV activity *ex vivo* on human serum samples by 37.2 ± 2.3 and 36.2 ± 2.6%, respectively, vs. plain RADA16. **p > 0.01.

In order to verify the stability and activity of HT hydrolysates and HP hydrolysates embedded in the RADA16 hydrogel, their DPPIV inhibitory activities were performed by *in situ* and *ex vivo* experiments. Before performing the *in situ* experiments, MTT experiments were performed in order to exclude any potential cytotoxic effect mediated by the new hydrogels on non-differentiated Caco-2 cells. These experiments demonstrated that they are safe at 1.0 mg mL^−1^, i.e., the concentration used for all biological experiments (Figure S2). After having assessed this important information, Caco-2 cells were seeded on the RADA16-HT and HP hydrogels (1.0 mg mL^−1^) and after 24 h their effects on the DPPIV activity were measured using AMC-Gly-Pro as a substrate (50 μM). Figure 4A shows that RADA16-HT and RADA16-HP hydrogels reduced the DPPIV activity by 38.3 ± 5.6 and 42.2 ± 3.0% vs. the RADA16 hydrogel, respectively. These findings indicate that the activities of the HT and HP hydrolysates are enhanced by 2.5- and 2.0-folds, respectively, when they are embedded in the hydrogel in respect to their plain solutions. This seems to indicate that the structuring of the HT and HP hydrolysates within the RADA16 hydrogel provides higher resistance toward the proteases that are expressed by Caco-2 cells. The RADA16-HT and RADA16-HP hydrogels represent dynamic systems in which some peptides actively contribute to the biomechanical, morphological, and structural properties of the nanoformulations, since transient electrostatic repulsions, between the hempseed peptides and the RADA16 molecules during self-assembly and nanofiber formation, can occur. These electrostatic interactions contribute to the stabilization of the fibrillar systems making them good carriers for bioactive compounds delivery. The experimental findings suggest that hempseed peptides, trapped inside the entangled nanofibrous domains of the hydrogels, are slowly released allowing their interaction with the DPPIV catalytic site. In fact, results pointed out that both HT and HP are released from the hydrogels as a function of time with a linear trend. In particular, using an experimental method proposed by Goa (1953) and already used by us (Lammi et al., 2014, 2016a), the released peptide concentrations were measured after 1, 3, and 6 h of incubation. Figure 4B shows that released HT peptide concentrations were 0.36 ± 0.06, 0.52 ± 0.15, and 0.92 ± 0.06 μg μL^−1^, whereas released HP concentrations were 0.23 ± 0.03, 0.41 ± 0.09, 0.79 ± 0.08 μg μL^−1^ after 1, 3, and 6 h, respectively. Overall, HT peptides were more delivered than HP peptides; this difference may be due to the different physical-chemical properties of each hempseed hydrolysate. As reported in Table S1 (see Supplementary Material), the HT hydrolysate shows a hydrophilicity of 63%, whereas HP peptides one of 57%. Being more hydrophobic, HP peptides are more retained within the shell of the hydrogel, whereas the more hydrophilic HT peptides are more released. Moreover, the HP hydrolysate contains longer peptides (>15 a.a. residues) than the HT hydrolysate. This explains why the HT peptides may more easily escape from the entangled nanofibrous domains of the hydrogels than HP peptides (Table S1). This slow release enhanced either the activity or the stability of both HT and HP hydrolysates.

This hypothesis was also confirmed by *ex vivo* experiments performed on human serum samples. More in detail, serum samples were incubated with either RADA16-HT or RADA16-HP hydrogels for 24 h at 37°C. Afterwards, the AMC-Gly-Pro substrate (50.0 μM) was added, and the fluorescence signals detected using a plate reader. Figure 4C indicates that both hydrogels are able to decrease the circulating DPPIV activity by 37.2 ± 2.3 and 36.2 ± 2.6%, respectively, vs. native RADA16. These results underline the enhanced stability that hempseed peptides acquire when they are embedded in the nanoformulations: in fact, both native hydrolysates were unable to inhibit the activity of circulating DPPIV in the *ex vivo* system probably due to their scarce capacity of resisting to the serum proteases activity.

### Synergistic Effect of RADA16-Hempseed Hydrolysates With Sitagliptin as DPPIV Inhibitors

**Figure 5:**
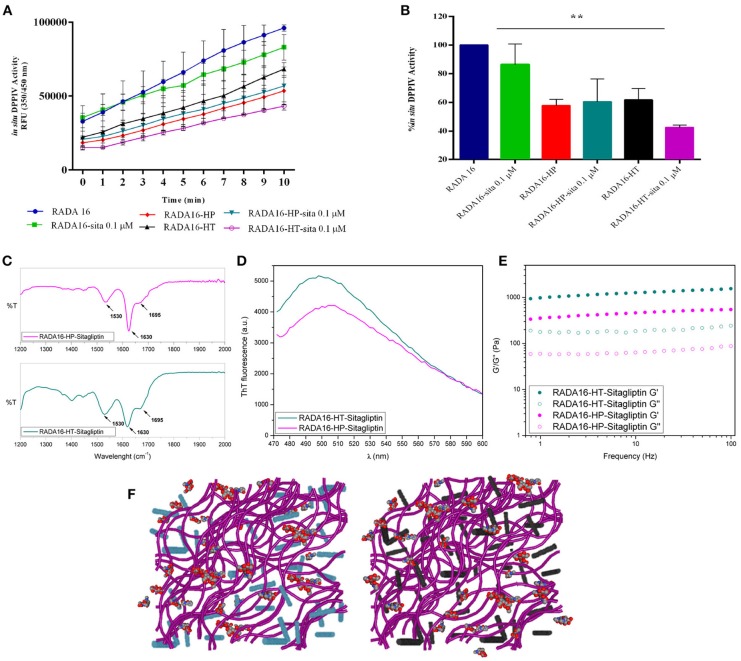
Synergistic effect of RADA16-hempseed hydrolysates with sitagliptin as DPPIV inhibitor in the Caco-2 cells. **(A)** Kinetics of the degradation of substrate Gly-Pro-AMC catalyzed by DPPIV. In respect to the control sample (RADA16-hydrogel), the DPPIV activity is slightly decreased when Caco-2 cells are incubated with RADA16 hydrogel containing 0.1 μM sitagliptin and it is much more reduced when cells are treated with either RADA16-HT or RADA16-HP hydrogels with or without 0.1 μM sitagliptin. **(B)** DPPIV activity after incubating Caco-2 cells with different hydrogels (at 5 min). The RADA16 hydrogel containing 0.1 μM sitagliptin reduced the DPPIV activity by 13.2 ± 8.3% vs. pure RADA16, whereas RADA16-HT and RADA16-HP alone drop the enzyme activity by 38.3 ± 5.6 and 42.2 ± 3.0%, respectively. RADA16-HT and RADA16-HP containing 0.1 μM sitagliptin reduced the enzyme activity by 56.4 ± 0.8 and 40.0 ± 9.1%, respectively. **(C,D)** Structural properties of RADA16-HT-sitagliptin (cyan) and RADA16-HP-sitagliptin (magenta) hydrogels. The ATR-FTIR structural analysis highlighted typical β-sheet signature in the Amide I and Amide II regions. ThT spectroscopy assay confirmed the cross-β-sheet conformation of both hydrogels in presence of sitagliptin. **(E)** Rheological characterization of RADA16-HT-sitagliptin and RADA16-HP-sitagliptin hydrogels monitored by frequency sweep tests (0.1–100 Hz) revealed elastic moduli of 1,028 Pa and 486 Pa, respectively. Both samples maintained viscoelastic profiles similar to those of RADA16-HT and RADA16-HP hydrogels along the tested frequency range at low strains (1%). **(F)** Cartoon models of RADA16-HT-Sitagliptin (left) and RADA16-HP-Sitagliptin (right) peptide based-hydrogels. RADA16 network of nanofibers is shown in purple, HT peptides in cyan, HP peptides in dark gray and sitagliptin is shown as spheres representation. **p > 0.01.

The last part of the experimentation was dedicated to investigating the possible synergist effects of combining sitagliptin with the RADA16-hempseed hydrogels (Figures 5A–F) for DPPIV inhibition. RADA16 hydrogels containing sitagliptin (at the final concentration of 0.1 μM) and RADA16 hempseed hydrogels containing sitagliptin (at the final concentration of 0.1 μM) were prepared and Caco-2 cells were seeded on the hydrogel. After 24 h, the spent medium was removed, cells were washed, the Gly-Pro-AMC substrate was added, and the fluorescence signals were monitored for 10 min. The results (Figures 5A,B) clearly indicate that at all reaction times, the DPPIV activity was the highest in the control samples treated with RADA16-hydrogel, slightly lower in cells incubated with RADA16 hydrogel containing 0.1 μM sitagliptin, and further reduced in cells incubated with RADA16-HT and RADA16-HP hydrogels with or without sitagliptin 0.1 μM. However, findings clearly suggest that only RADA16-HT containing 0.1 μM sitagliptin shows a synergistic inhibitory effect of DPPIV, which started 3 min after the addition of the Gly-Pro-AMC substrate and remained constant for up to 10 min of incubation (Figure 5B). The reaction rate followed a linear trend and 5 min of incubation with Gly-Pro-AMC corresponded to one half of the linear tract. Figure 5B shows the results at 5 min. RADA16 hydrogel containing 0.1 μM sitagliptin reduced the DPPIV activity by 13.2 ± 8.3% vs. RADA16 alone, whereas the inhibition of the enzyme activity reached 38.3 ± 5.6 and 42.2 ± 3.0%, respectively, incubating with control RADA16-HT and RADA16-HP hydrogels, and 56.4 ± 0.8 and 40.0 ± 9.1%, respectively, incubating with RADA16-HT and RADA16-HP hydrogels containing 0.1 μM sitagliptin.

From a nanostructural point of view, the addition of sitagliptin did not affect the physicochemical properties of the RADA16-HT and RADA16-HP hydrogels. The ATR-FTIR structural analysis highlighted typical β-sheet signatures in the Amide I and Amide II regions (Figure 5C) and the ThT spectroscopy assay confirmed the cross-β-sheet conformation of both hydrogels in the presence of sitagliptin (Figure 5D). In the same way, frequency sweep tests of the resulting RADA16-HP-sitagliptin and RADA16-HT-sitagliptin hydrogels revealed elastic moduli of 486 and 1,028 Pa, respectively (Figure 5E), indicating that they still maintain viscoelastic profiles similar to those of RADA16-HT and RADA16-HP samples. Lastly, an insight of the RADA16 nanofibers bundles formation trapped with hempseed peptides and sitagliptin is provided through a cartoon model as depicted on Figure 5F.

In summary, these results underline one important aspect, namely that RADA16-hempseed hydrolysates hydrogels are not only good sitagliptin delivery carriers, but are also active as DPPIV inhibitors suggesting that the combination of sitagliptin with food protein based hydrogels may help reducing drug dosage and their potential side-effects.

## Conclusion

This work offers a new route for the formulation of nano-nutraceuticals entirely made of biocompatible/bioabsorbable SAP based-hydrogels and bioactive hydrolysates to be exploited in diabetes and metabolic diseases prevention.

## Methods

### Materials

Fmoc-Arg(Pbf)-OH, Fmoc-Ala-OH, and Fmoc-Asp(OtBu)-OH were purchased from the Aapptec (Louisville, USA) and used as received. N,N-dimethylformamide (DMF), N,N-diisopropylethylamine (DIPEA), N-methyl-2-pyrrolidone (NMP), trifluoroacetic acid (TFA) and triisopropylsilane were purchased from VWR (Radnor, USA). N,N,N′,N′-tetramethyl-O-(1H-benzotriazol-1-yl)uronium hexafluorophosphate (HBTU), 1-hydroxybenzotriazole hydrate (HoBT), 4-metylpiperidin and Thioflavin T were purchased from Sigma-Aldrich. HPLC grade water (resistivity 18 MΩ cm) and DPBS (pH 7.4) were purchased from Thermo Fisher scientific (Waltham, USA).

### Production of HT and HP Hydrolysates

HT and HP hydrolysates were obtained extracting the proteins from the seeds of *Cannabis sativa* cultivar Futura, by hydrolyzing them with pepsin or trypsin and by analyzing them as described elsewhere (Aiello et al., 2017; Zanoni et al., 2017).

### *In vitro* DPPIV Activity Assay

The experiments were carried out using a procedure previously reported (Lammi et al., 2018). Briefly, 0.5 and 1.0 mg mL^−1^ of HT and HP hydrolysates were tested *in vitro* using the purified recombinant DPP-IV enzyme and fluorescent substrate (AMC-Gly-Pro, ex/em 360/465 nm). Fluorescence signals were measured using the Synergy H1 from Biotek (Bad Friedrichshall, Germany). More details are reported in the Supplementary Materials.

### Cell Culture

Caco-2 cells, obtained from INSERM (Paris), were cultured at 50% density following the procedure previously reported (Lammi et al., 2018). More details are reported in the Supplementary Materials.

### *In situ* DPPIV Activity Assay

HT and HP hydrolysates were tested on Caco-2 cells (5 × 10^4^/well in black 96-well plates) at 1.0 mg mL^−1^ or vehicle in growth medium for 24 h at 37°C, following the method previously optimized and reported (Lammi et al., 2018). For 2D cell culture on RADA16-HT and RADA-HP hydrogels, Caco-2 cells were seeded on the surface of the above mentioned hydrogels at the density of 5 × 10^4^/well; on the day after the spent media were removed and cells were washed with 100 μL of PBS without Ca^++^ and Mg^++^, and 100 μL of DPPIV substrate at the concentration of 50.0 μM in PBS without Ca^++^ and Mg^++^ were added in each well. Fluorescence signals (ex./em. 350/450 nm) were measured using the Synergy H1 from Biotek every 1 min for 10 min.

### *Ex vivo* DPP-IV Activity Assay

A volume of 40 μL of serum samples was loaded in each well of the black 96-well plates and 10 μL of the 5 × HT and HP hydrolysates were spiked in order to have the final concentration of 1.0 mg mL^−1^ in the total volume of 50 μL. For hydrogel experiments, 40 μL of human serum samples were incubated with RADA16-HT and RADA-HP. Samples were then incubated for 24 h at 37°C. Subsequently, 50 μL of the AMC-Gly-Pro at the initial concentration of 100 μM were added in each well in order to obtain the final 50 μM substrate concentration in the final volume of 100 μL. Fluorescence signals (ex./em. 350/450 nm) were measured using the Synergy H1 every 1 min for 10 min.

### Determination of HT and HP Peptides Release From the Hydrogels

The peptide leaking from the hydrogels as a function of time was measured according to a method previously described (Goa, 1953; Lammi et al., 2016a). Briefly, a sterile solution of peptone from casein at 10 mg mL^−1^ in water was prepared and used as standard for the calibration curves. Thus, a solution of X μL of HT and HP peptides contained in the hydrogels after 1, 3, and 6 h of incubation and/or peptone mixture, (100 – X) μL water, 95 μL 6% (w/w) NaOH in water, and 9.5 μL of active reagent (containing 0.6 M sodium citrate, 0.9 M sodium carbonate, and 0.07 M copper sulfate, 2.4 M NaOH, pH 10.6) was prepared. The reaction mixture was incubated for 15 min at RT and the absorbance was measured at 330 nm using the Synergy H1 plate reader.

### Peptide Synthesis and Purification

RADA16 was synthesized by solid-phase Fmoc-based chemistry on Rink amide 4-methyl-benzhydrylamine resin (0.5 mmol g^−1^ substitution) using the Liberty-Discovery (CEM) microwave automated synthesizer (Matthews, USA), as previously described (Gelain et al., 2010).

### Assembly of RADA16 Embedded With Hemp-Protein Hydrolysates

The purified RADA16 was dissolved at 10 mg mL^−1^ in distilled water, sonicated for 30 min, and incubated at 4°C for 24 h. HT and HP hydrolysates were dissolved at 1.0 mg mL^−1^ in distilled water. Sitagliptin was used at a final concentration of 0.1 μM. The RADA16 solution was then mixed with HT and HP solutions at a ratio of 3:1 (v/v), whereas RADA16 was mixed with the solution containing both hempseed hydrolysates and sitagliptin at a final ratio of 3:0.5:0.5 (v/v).

### Spectroscopic Analysis

FT-IR analysis of assembled nanostructures was performed on peptides dissolved at a concentration of 1% (w/v) in distilled water, after 24 h incubation at 4°C. All spectra were collected in attenuated total reflection (ATR) using Perkin Elmer Spectrum 100 spectrometer. All the collected spectra were reported after ATR correction, smoothing, and automatic baseline correction using Origin^TM^8 software. Each sample was analyzed in triplicate. In order to assess the presence of cross-β fibril structures, ThT binding assay was monitored by exciting the sample at 440 nm (5 nm band-pass) and recording the emission fluorescence spectrum from 460 to 600 nm.

### Rheological Tests

Rheological properties of assembled nanostructures were carried out using a controlled stress AR-2000ex Rheometer (TA instruments). A truncated cone-plate geometry (acrylic truncated diameter, 20 mm; angle, 1°; truncation gap, 34 μm) was used. All measurements were obtained at 25°C using a Peltier cell as a lower plate of the instrument to keep the temperature controlled during each test. All samples were tested 1day after dissolution at the concentration of 1% (w/v). Frequency sweep experiments were recorded as a function angular frequency (0.1–100 Hz) at a fixed strain of 1%. Strain sweeps were performed on samples from 0.01% to a maximum strain of 1,000% for determining the limit of the linear viscoelastic region and the maximum strain to which the sample can be subjected. Each experiment was performed in triplicate.

### Atomic Force Microscopy

AFM tests were performed in tapping mode by a Multimode Nanoscope V (Digital Instrument, Veeco), using a single-beam silicon cantilever probes (Veeco RFESP MPP-21100-10, cantilever f0, resonance frequency 59–69 KHz, constant force 3 N m^−1^), as previously described (Pugliese et al., 2018a).

### Statistical Analysis

Statistical analyses were carried out by t-student and One-way ANOVA using Graphpad Prism 6 (Graphpad, La Jolla, CA, USA) followed by Dunnett's test. Values were expressed as means ± SD; *P* < 0.05 were considered to be significant.

## Author Contributions

CL and RP conceived the project and designed the experiments. CL performed all *in vitro, in situ*, and *ex vivo* tests and the preparation of hempseed hydrolysates. CB performed technical work in the bioactivity characterization. RP synthesized the RADA16 peptide and carried out all structural, morphological, and biomechanical experiments. FG co-supervised the SAP characterizations. CL, AA, and RP wrote the manuscript.

### Conflict of Interest Statement

The authors declare that the research was conducted in the absence of any commercial or financial relationships that could be construed as a potential conflict of interest.
