# Supplementary material for: Enhancement of the Stability and Anti-DPPIV Activity of Hempseed Hydrolysates Through Self-Assembling Peptide-Based Hydrogels
Source: Front Chem. 2019 Jan 24;6:670. doi: 10.3389/fchem.2018.00670 (PMC6353790; doi:10.3389/fchem.2018.00670)
Supplement: Supplementary file 1 [file Data_Sheet_1.docx]

Supplementary Material

**Enhancement of the stability and anti-DPPIV activity of hempseed hydrolysates through self-assembling peptide-based hydrogels**

**Carmen Lammi^1,^,*^, Carlotta Bollati^1^, Fabrizio Gelain^2,3^, Anna Arnoldi^1^, Raffaele Pugliese^2,3,^^**

^1^Department of Pharmaceutical Sciences, University of Milan, Milan, Italy

^2^Tissue Engineering Unit, Institute for Stem Cell Biology, Regenerative Medicine and Innovative Therapies-ISBReMIT, Fondazione IRCSS Casa Sollievo della Sofferenza, San Giovanni Rotondo (FG), Italy

^3^Center for Nanomedicine and Tissue Engineering (CNTE), ASST Grande Ospedale Metropolitano Niguarda, Milan, Italy

*** Correspondence:**Carmen Lammi: [carmen.lammi@unimi.it](mailto:carmen.lammi@unimi.it)

^These authors equally contributed to this work.

**Methods**

**In vitro DPPIV activity assay**. The DPPIV enzyme and the substrate solution (5 mM H-Gly-Pro-AMC) were provided by Cayman Chemicals (Michigan, USA). The experiments were carried out in triplicate in a half volume 96-well solid plate (white). Each reaction (50 µL) was prepared adding the reagents in a microcentrifuge tube in the following order: 1 X assay buffer [20 mM Tris-HCl, pH 8.0, containing 100 mM NaCl, and 1 mM EDTA] (30 µL), 0.5 and 1.0 mg mL^-1^ of HT and HP hydrolysates or vehicle (10 µL) and finally the DPP IV enzyme (10 µL). Subsequently, the samples were mixed and 50 µL of each reaction were transferred in each plate well. Each reaction was started by adding 50 µL of substrate solution to each well and incubated at 37 °C for 1 h. Fluorescence signals were measured using the Synergy H1 fluorescent plate reader from Biotek (excitation and emission wavelengths 360 and 465 nm, respectively).

**Cell culture.** Caco-2 cells, obtained from INSERM (Paris), were routinely sub-cultured at 50% density and were maintained at 37 °C in a 90% air–10% CO2 atmosphere in Dulbecco Minimum Essential Medium (DMEM) containing 25 mM glucose, 3.7 g/L NaHCO3, 4 mM stable L-glutamine, 1% nonessential amino acids, 100 U/L penicillin, 100 µg/L streptomycin (complete medium), supplemented with 10% heat-inactivated fetal bovine serum (FBS Hyclone Laboratories, Logan, UT, USA).

***In situ* DPPIV activity assay.** A total of 5×10^4^/well Caco-2 cells were seeded in black 96-well plates with clear bottom. The second day after seeding, spent media was discarded and Caco-2 cells were treated with HT and HP hydrolysates at 1.0 mg mL^-1^ or vehicle in growth medium for 24 h at 37 °C. Afterwards, treatments were removed and Caco-2 cells washed once with 100 µL of PBS w/o Ca^++^ and Mg^++^. Thus, 100 µL of AMC-Gly-Pro substrate were added in each well at the concentration of 50.0 µM in PBS without Ca^++^ and Mg^++.^ For 2D cell culture on RADA16-HT and RADA-HP hydrogels, Caco-2 cells were seeded on the surface of the above mentioned hydrogels at the density of 5×10^4^/well; on the day after the spent media were removed and cells were washed with 100 µL of PBS without Ca^++^ and Mg^++^, and 100 µL of DPPIV substrate at the concentration of 50.0 µM in PBS without Ca^++^ and Mg^++^ were added in each well. Fluorescence signals (ex./em. 350/450 nm) were measured using the Synergy H1 from Biotek (Bad Friedrichshall, Germany) every 1 min for 10 min.

**Peptide synthesis and purification.** Synthesis was carried out with 0.5 M HBTU in DMF and 2 M DPIEA in NMP as activator and activator base solutions respectively. Fmoc-protected amino acids were dissolved at 0.2 M in DMF. Fmoc-groups were removed by using a 20% (v/v) solution of 4-methylpiperidin in DMF. Peptide side chains removal and cleavage were performed with TFA:TIS:H2O (95:2.5:2.5) cocktail. The C-terminal of peptide sequence was amidated after the cleavage and the N-terminal was acetylated using a 20% v/v solution of acetic anhydride in DMF. The raw peptide was precipitated using cold ethyl ether and then lyophilized. The resulting raw peptide was purified using a Waters binary high-performance liquid chromatography (HPLC) apparatus (>95%) on a Restek^TM^ Prep C18 column, using a gradient of acetonitrile in H_2_O with 0.1% TFA over 30 min. The molecular weight of purified peptide was identified via single quadrupole mass detection (Waters LC-MS Alliance-3100). Purified peptide powder was subsequently dissolved in 0.1 M HCl solution in order to remove all TFA salts.

**Spectroscopic analysis.** Aliquots of 2 μL of each peptide solution were deposited on the reflection diamond element of the ATR device and the spectra were recorded after solvent evaporation. Twenty acquisitions were recorded for each spectrum, using the following condition: 4 cm^-1^ spectrum resolution, 25 kHz scan speed, 1000 scan co-addition, and a triangular apodization. All the collected spectra were reported after ATR correction, smoothing, and automatic baseline correction using Origin^TM^8 software. Each sample was analyzed in triplicate.

Thioflavin T molecule consists of a benzylamine and a benzathiole ring connected through a carbon-carbon bond. These two rings can rotate freely when the molecule is in solution. However, when ThT binds to cross-beta fibrils, the two rotational planes of the two rings become immobilized and therefore, this molecule can maintain its excited state resulting in a high quantum yield of fluorescence. ThT stock solution was prepared by adding 8 mg of ThT to 10 mL phosphate buffer (10 mM phosphate, 150 mM NaCl, pH 7) and filtered through a 0.2 μm syringe filter. Right before the analysis, 1 mL of ThT stock solution was diluted into 50 mL of phosphate buffer (working solution). Peptides at 1% (w/v) were mixed with working solution (1:0.5 v/v) and stirred for 2 min.

**Atomic force microscopy.** All peptides were dissolved in distilled water on the day prior to imaging; right before the analysis, peptides solutions were diluted to a final concentration of 0.001% (w/v) and deposited on a freshly cleaved mica surface. A 2 μL of each solution was kept on mica for 4 min at RT, subsequently rinsed with distilled water (to remove loosely bound peptides), and dried at RT for 30 min when it was imaged.

**Table S1. LC-ESI-MS/MS based identification of hydrolysates of hempseed proteins.**

| **Accession n. *^a^*** | **Protein Name** | **Start-end** | **Sequences** | **pI*^b^*** | **Net Charge *^b^*** | | **Hydrophobicity % *^c^*** |
| --- | --- | --- | --- | --- | --- | --- | --- |
| ***^*^Peptic*** |  |  |  |  |  | |  |
| A0A090CXP8 | Edestin 2 | 176-187 | (D)WVYNNGDSPLVL(I) | 0.7 | -1 | 50 | |
|  |  | 361-370 | (V)LYKNGMMAPH(F) | 9.7 | 1.1 | 50 | |
|  |  | 380-403 | (I)YVTRGSARLQVVDDNGRNVFDGEL(R) | 4.3 | -1 | 33.3 | |
|  |  | 435-447 | (N)DNAMRNPLAGKVS(A) | 10.2 | 1 | 46.2 | |
|  |  | 235-247 | (R)RESGEQTPNGNIF(S) | 4.2 | -1 | 23.1 | |
| A0A090CXP7 | Edestin 1 | 450-460 | (A)WVSPLAGRTSV(I) | 10.7 | 1 | 54.6 | |
|  |  | 178-187 | (L)LDTSNVNNQL(D) | 0.7 | -1 | 30 | |
|  |  | 279-288 | (D)LVSPLRSSQE(H) | 6.9 | 0 | 40 | |
|  |  | 63-73 | (L)IESWNPNHNQF(Q) | 5.1 | -0.9 | 36.4 | |
|  |  | 461-469 | (V)IRALPEAVL(A) | 6.9 | 0 | 77.8 | |
| A0A090DLH8 | Edestin 1 | 392-409 | (M)YVLRGRARVQVVNHMGQKC(F) | 11.2 | 4 | 36.8 | |
| H9A1V3 | Acyl-activating enzyme 1 | 625-639 | (I)ERVCNEVDDRVFETT(A) | 3.8 | -3.1 | 26.7 | |
|  |  | 170-190 | (G)GYLNSAKNCLNVNSNKKLNDT(M) | 9.6 | 1.9 | 23.8 | |
| H9A1V4 | Acyl-activating enzyme 2 | 280-298 | (H)IFDRVIEELFILHGASIGF(W) | 4.3 | -1.9 | 57.9 | |
|  |  | 28-44 | (Y)RSMYAKDGFPPPIDGLD(C) | 4.0 | -1 | 47.1 | |
|  |  | 442-453 | (G)PPVPNVDVCLES(V) | 0.7 | -2.1 | 58.3 | |
| H9A1V5 | Acyl-activating enzyme 3 | 293-312 | (L)ALSKNSMVKKFNLSSIKYIG(S) | 10.8 | 4 | 40 | |
|  |  | 360-382 | (N)SGSAGMLASGVEAQIVSVDTLKP(L) | 3.9 | -1 | 47.8 | |
| H9A1V7 | Acyl-activating enzyme 5 | 253-266 | (G)YTWGTAAVGATNVC(L) | 3.1 | -0.1 | 42.9 | |
|  |  | 497-512 | (F)VTLKKGAVRVTVTEKE(I) | 10.4 | 2 | 37.5 | |
|  |  | 54-62 | (T)RCLRVASCI(E) | 8.8 | 1.9 | 44.4 | |
| H9A1W0 | Acyl-activating enzyme 8 | 352-373 | (D)QNGSAQLAGVSGEVCIRGPNVT(K) | 6.1 | -0.1 | 36.4 | |
|  |  | 166-189 | (D)VALFLHTSGTTSRPKGVPLTQLNL(A) | 11.4 | 2.1 | 45.8 | |
| H9A1W2 | Acyl-activating enzyme 10 | 138-154 | (Q)NIAAKTSAQFSLIPSVP(S) | 9.7 | 1 | 58.8 | |
| H9A1W3 | Acyl-activating enzyme 11 | 260-268 | (F)EMKKMVELI(E) | 7.0 | 0 | 55.6 | |
|  |  | 8-14 | (F)IFRSKLP(D) | 11.4 | 2 | 57.1 | |
| H9A8L2 | Acyl-activating enzyme 13 | 160-180 | (P)GAVLNIAECCLLPTSYPRKDD(D) | 4.2 | -1.1 | 42.9 | |
|  |  | 289-309 | (P)LYSRVVEAAPDRVIVLPATGS(N) | 6.9 | 0 | 57.1 | |
|  |  | 535-547 | (Y)PDDQACTGEVGLI(P) | 0.5 | -3.1 | 38.5 | |
| H9A8L3 | Acyl-activating enzyme 14 | 374-392 | (A)IPWTQLSPIRCAAESWAHM(D) | 7.1 | 0 | 57.9 | |
|  |  | 598-615 | (I)KRTVGGYFIVQGRADDTM(N) | 9.5 | 1 | 33.3 | |
|  |  | 631-652 | (V)CDRADESIVETAAVSVSPVDGG(P) | 3.3 | -4.1 | 40.9 | |
| A7IZZ2 | (+)-alpha-pinene synthase, chloroplastic | 270-283 | (I)RAEAKWFIEEYEKT(Q) | 4.6 | -1 | 35.7 | |
|  |  | 592-606 | (G)DGHASQDSHSRKRIS(D) | 10.1 | 1.2 | 13.3 | |
|  |  | 319-336 | (H)SELGKNKMVYARDRLVEA(F) | 9.4 | 1 | 38.9 | |
|  |  | 185-201 | (I)FNDFKDETGKFKASIKN(D) | 9.5 | 1 | 29.4 | |
| A0A0C5ARX6 | ATP synthase subunit alpha | 123-131 | (I)STSESRLIE(S) | 4.2 | -1 | 22.2 | |
|  |  | 134-154 | (P)APGIISRRSVYEPLQTGLIAI(D) | 9.9 | 1 | 52.4 | |
| A0A0C5ARS5 | ATP synthase subunit beta | 382-405 | (G)EEHYETAQRVKQTLQRYKELQDII(A) | 5.5 | -0.9 | 25 | |
|  |  | 144-158 | (D)TKLSIFETGIKVVDL(L) | 6.6 | 0 | 46.7 | |
| E5DK51 | ATP synthase subunit alpha | 151-166 | (E)TLYCVYVAIGQKRSTV(A) | 9.4 | 1.9 | 37.5 | |
|  |  | 287-309 | (D)VSAYIPTNVISITDGQICLETEL(F) | 0.6 | -3.1 | 43.5 | |
| A6P6W0 | Cannabidiolic acid synthase-like 1 | 504-522 | (A)RIWGEKYFGKNFNRLVKVK(T) | 11.1 | 5 | 36.8 | |
|  |  | 91-104 | (V)SHIQGTILCSKKVG(L) | 9.7 | 2 | 28.6 | |
| A0A088MFF4 | Delta 12 desaturase | 179-196 | (P)PGRVLSLFVTLTLGWPLY(L) | 10.3 | 1 | 61.1 | |
|  |  | 331-345 | (Y)NAMEATKAVKPILGE(Y) | 6.6 | 0 | 53.3 | |
| A0A0C5ARQ8 | RNA polymerase subunit beta | 1047-1063 | (L)RSLALELNHFLVSEKNF(Q) | 7.5 | 0.1 | 47.1 | |
|  |  | 549-568 | (M)QRQAVPLSRSEKCIVGTGLE(S) | 8.6 | 0.9 | 35 | |
|  |  | 743-752 | (L)TPQMAKESSY(A) | 6.5 | 0 | 30 | |
|  |  | 358-377 | (T)STTLTTTFESFFGLHPLSQV(L) | 5.1 | -0.9 | 40 | |
|  |  | 17-26 | (N)QIQFEGFCRF(I) | 6.1 | -0.1 | 40 | |
| A0A0C5AS14 | Hypothetical chloroplast RF1 | 341-355 | (Q)ENSKLEILNEKKGVN(Y) | 7.1 | 0 | 26.7 | |
|  |  | 259-279 | (T)DVEIETTSETKGTKQEQGGST(E) | 3.9 | -3 | 9.5 | |
| A7IZZ1 | (-)-limonene synthase, chloroplastic | 180-200 | (L)RQYGFEVPQEIFNNFKNHKTG(E) | 9.4 | 1.1 | 28.6 | |
|  |  | 349-360 | (G)VRFEPQFSYFRI(M) | 9.8 | 1 | 50 | |
| E5DKP2 | MatR | 382-400 | (G)VQLAETLGTAGVRGPQVSV(L) | 6.8 | 0 | 47.4 | |
|  |  | 242-250 | (R)KLAAPLKTH(Y) | 10.7 | 2.1 | 55.6 | |
| A0A0C5AUJ6 | NADH-plastoquinone oxidoreductase subunit 5 | 603-622 | (M)DWNWYEFLTNATFSVSIASL(G) | 0.6 | -2 | 50 | |
|  |  | 256-269 | (E)GPTPISALIHAATM(V) | 7.8 | 0.10 | 64.3 | |
| A0A0C5APZ1 | NAD(P)H-quinone oxidoreductase chain 4 | 234-257 | (W)LPDTHGEAHYSTCMLLAGILLKMG(A) | 6.1 | -0.9 | 45.8 | |
|  |  | 230-238 | (P)LHTWLPDTH(G) | 6.0 | -0.8 | 44.4 | |
| Q8RVK9 | Naringenin-chalcone synthase | 353-368 | (K)CVEDGLNTTGEGLEWG(V) | 0.5 | -4.1 | 25 | |
|  |  | 301-324 | (W)IAHPGGPAILDQVESKLALKTEKL(R) | 7.8 | 0.1 | 50 | |
|  |  | 236-250 | (P)IFELVSAAQTILPDS(D) | 0.7 | -2 | 60 | |
|  |  | 183-201 | (K)GARVLVVCSEITAVTFRGP(N) | 8.9 | 0.9 | 52.6 | |
| V5KXG5 | 4-coumarate:CoA ligase | 262-281 | (G)ATILIMPKFEIGSLLGLIER(Y) | 7.1 | 0 | 60 | |
|  |  | 17-23 | (I)IFRSKLP(D) | 11.4 | 2 | 57.1 | |
| F1LKH7 | Polyketide synthase 2 | 371-385 | (G)LTVERVVLRSVPINY(-) | 9.8 | 1 | 53.3 | |
|  |  | 303-310 | (A)ILDKVEEK(L) | 4.3 | -1 | 37.5 | |
| F1LKH8 | Polyketide synthase 4 | 2-16 | (M)NHLRAEGPASVLAIG(T) | 7.4 | 0.1 | 53.3 | |
|  |  | 256-266 | (A)GLIFDLHKDVP(M) | 5.0 | -0.9 | 54.6 | |
| A0A0C5APZ4 | Protein Ycf2 | 1630-1650 | (P)FSLRLALSLSRGILVIGSIGT(G) | 12.1 | 2 | 52.4 | |
|  |  | 1902-1921 | (Q)DHGILFYQIGRAVAQNVLLS(N) | 7.8 | 0.1 | 50 | |
|  |  | 1092-1102 | (T)ISPIELQVSNI(F) | 0.9 | -1 | 54.6 | |
|  |  | 536-547 | (S)ENKEIVNIFKII(T) | 7.0 | 0 | 50 | |
|  |  | 143-152 | (L)YLPKGKKISE(S) | 10.1 | 2 | 30 | |
| A0A0C5ARZ4 | Photosystem I P700 chlorophyll a apoprotein A1 | 436-454 | (I)SHLNWVCIFLGFHSFGLYI(H) | 7.2 | 0.1 | 52.6 | |
|  |  | 102-122 | (W)LSDPTHIGPSAQVVWPIVGQE(I) | 3.9 | -1.9 | 52.4 | |
|  |  | 561-572 | (L)IPDKANLGFRFP(C) | 10.1 | 1 | 58.3 | |
| A0A0C5APY0 | Photosystem I P700 chlorophyll a apoprotein A2 | 188-207 | (S)LAWTGHLVHVAIPGSRGESV(R) | 8.0 | 0.2 | 50 | |
|  |  | 247-257 | (T)SQGAGTSILTL(L) | 3.4 | 0 | 36.4 | |
|  |  | 241-258 | (S)SHLFGTSQGAGTSILTLL(G) | 7.5 | 0.1 | 38.9 | |
|  |  | 352-369 | (H)MYSLPAYAFIAQDFTTQA(A) | 0.7 | -1 | 55.6 | |
|  |  | 695-708 | (R)DKPVALSIVQARLV(G) | 10.2 | 1 | 64.3 | |
| U6EFF4 | Putative LysM domain containing receptor kinase | 398-417 | (H)LRGSGRDPLTWSSRVQIALD(S) | 10.5 | 1 | 40 | |
|  |  | 125-144 | (V)HRVNMFKPTRIPAGSPINVT(V) | 12.1 | 3.1 | 50 | |
|  |  | 115-142 | (A)FANLTTEDWVHRVNMFKPTRIPAGSPIN(V) | 9.9 | 1.1 | 50 | |
| A0A0E3TIL1 | THCA synthase | 87-102 | (T)PSNNSHIQATILCSKK(V) | 10.3 | 2 | 31.3 | |
|  |  | 29-47 | (A)NPRENFLKCFSKHIPNNVA(N) | 9.6 | 2 | 42.1 | |
|  |  | 504-522 | (A)RIWGEKYFGKNFNRLVKVK(T) | 11.1 | 5 | 36.8 | |
| ***^**^Tryptic*** |  |  |  |  |  |  | |
| A0A090CXP7 | Edestin 1 | 245-258 | (R)YLEEAFNVDSETVK(R) | 3.5 | -3 | 35.7 | |
|  |  | 194-208 | (R)FYLAGNPEDEFEQLR(R) | 3.5 | -3 | 40 | |
|  |  | 275-284 | (K)GTLDLVSPLR(S) | 6.6 | 0 | 50 | |
|  |  | 417-431 | (R)QGQIVTVPQNHAVVK(Q) | 9.84 | 1.1 | 46.7 | |
|  |  | 176-192 | (V)SLLDTSNVNNQLDDNPR(R) | 3.4 | -2 | 29.4 | |
|  |  | 198-208 | (A)GNPEDEFEQLR(R) | 3.5 | -3 | 27.3 | |
|  |  | 485-504 | (K)YNREETVLLTSSTSSRREDR(Y) | 6.84 | 0 | 15 | |
|  |  | 56-73 | (R)VEAEAGLIESWNPNHNQF(Q) | 3.8 | -2.9 | 44.4 | |
|  |  | 246-258 | (Y)LEEAFNVDSETVK(R) | 3.54 | -3 | 38.5 | |
|  |  | 150-160 | (R)EGDIVAIPAGV(A) | 0.7 | -2 | 63.6 | |
| A0A090CXP8 | Edestin 2 | 284-294 | (R)GEDLQIIAPSR(I) | 3.9 | -1 | 45.5 | |
|  |  | 320-334 | (R)QNIDRPSQADIFNPR(G) | 6.5 | 0 | 40 | |
|  |  | 397-404 | (R)NVFDGELR(E) | 3.9 | -1 | 37.5 | |
|  |  | 256-268 | (L)AESFNVDTELAHK(L) | 4.3 | -1.9 | 38.5 | |
|  |  | 163-176 | (K)EGDMVAMPAGVADW(V) | 0.48 | -3 | 64.3 | |
|  |  | 427-439 | (F)EWIAVKTNDNAMR(N) | 6.9 | 0 | 46.2 | |
| A0A0C5ARX1 | Photosystem II protein D1 | 196-222 | (H)PFHMLGVAGVFGGSLFSAMHGSLVTSS(L) | 8.4 | 0.2 | 51.9 | |
|  |  | 64-92 | (I)REPVSGSLLYGNNIISGAIIPTSAAIGLH(F) | 7.51 | 0.1 | 48.3 | |
|  |  | 332-350 | (M)HERNAHNFPLDLAALEVPS(T) | 5.1 | -1.8 | 52.6 | |
| H9A8L3 | Acyl-activating enzyme 14 | 708-722 | (K)LLRRVLRDQIKHELS(A) | 11.12 | 2.1 | 40 | |
|  |  | 214-240 | (T)LKQLREQVISVAKALDAMFSKGDAIAI(D) | 9.8 | 1 | 55.6 | |
| H9A1W0 | Acyl-activating enzyme 8 | 412-425 | (R)IKELINRGGEKISP(I) | 9.9 | 1 | 35.7 | |
|  |  | 424-440 | (I)SPIEVDAVLLSHPDISH(A) | 4.2 | -2.8 | 52.9 | |
| E5DKP2 | MatR | 386-411 | (A)ETLGTAGVRGPQVSVLWGTVKHIRQG(S) | 11.2 | 2.1 | 38.5 | |
|  |  | 38-59 | (F)YSIQKVFSAGRLVGGEKGPYSV(P) | 9.9 | 2 | 36.4 | |

1. According to “UniProtKB” (<http://www.uniprot.org/>)
2. According to “Protein Peptide Calculator” (<http://pepcalc.com/>)
3. According to “Peptide2.0” (<http://peptide2.com/>)

*(Zanoni, Aiello, Arnoldi, & Lammi, 2017)

**(Aiello, Lammi, Boschin, Zanoni, & Arnoldi, 2017)


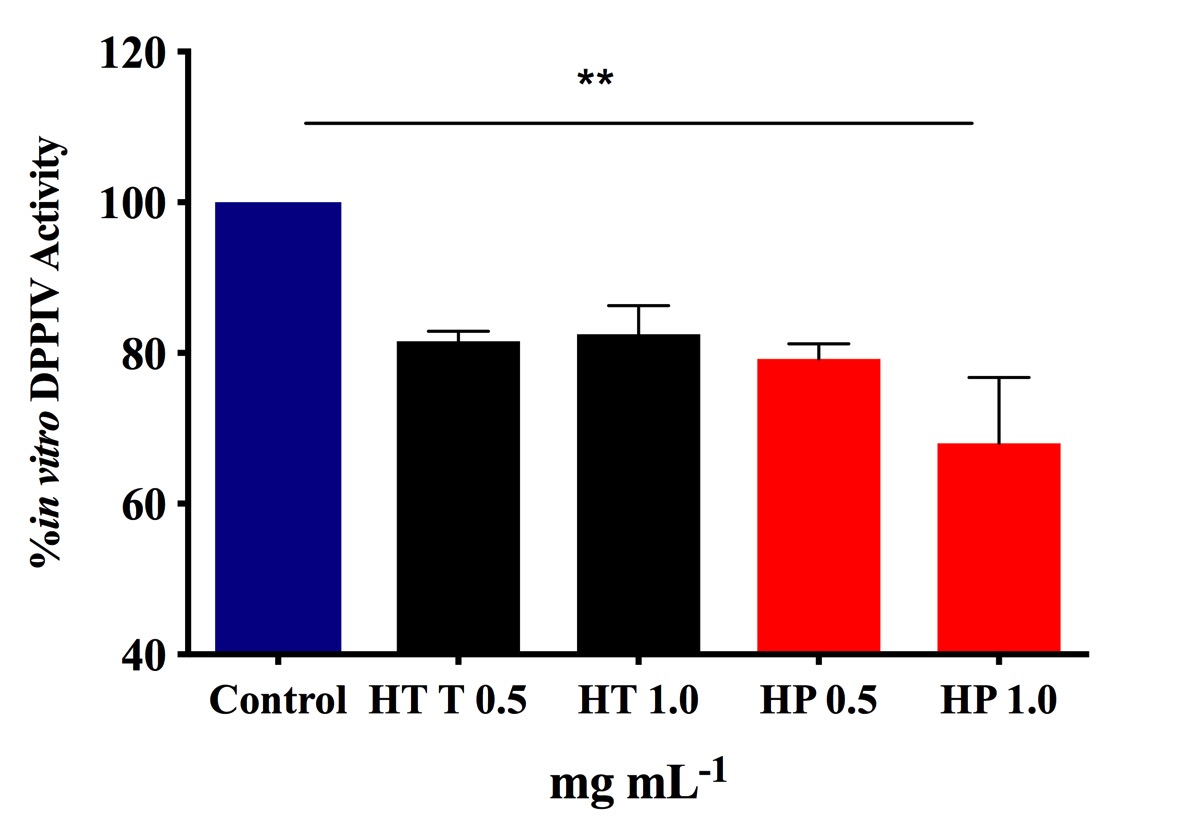


**Figure S1. Inhibition of DPPIV activity by HT and HP hydrolysates.** HT inhibits *in vitro* the activity of human recombinant DPPIV by 18.5±1.3% and 17.5±2.7% whereas HP hydrolysates by 20.8±1.3 and 32.0±6.2%, at 0.5 and 1.0 mg mL^-1^, respectively


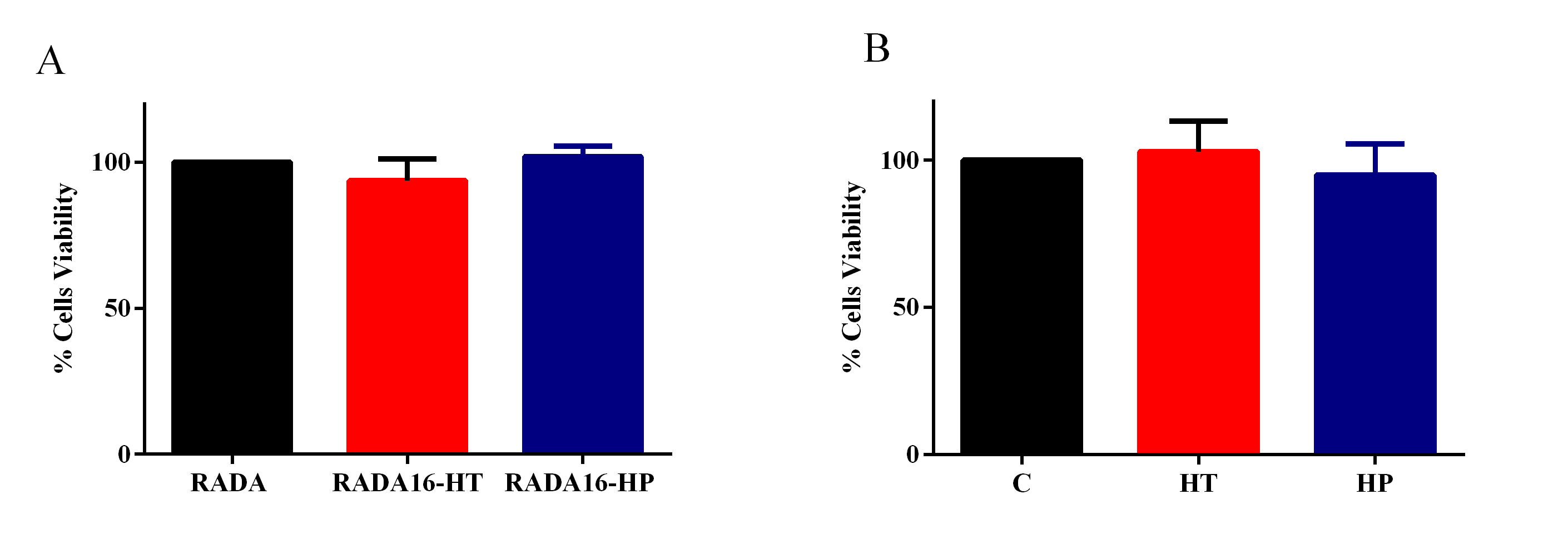


**Figure S2.** MTT results indicate that RADA16-HT, RADA16-HP, HT, and HP, tested at the concnetration of 1.0 mg mL^-1^ for 48 h, did not induce CaCo-2 cell mortality. Data points represent averages ± s.d. of three independent experiments in duplicate.

**References**

Aiello, G., Lammi, C., Boschin, G., Zanoni, C., & Arnoldi, A. (2017). Exploration of Potentially Bioactive Peptides Generated from the Enzymatic Hydrolysis of Hempseed Proteins. *Journal of Agricultural and Food Chemistry, 65*(47), 10174-10184.

Zanoni, C., Aiello, G., Arnoldi, A., & Lammi, C. (2017). Hempseed peptides exert hypocholesterolemic effects with a statin-like mechanism. *J Agric Food Chem, 65*(40), 8829-8838.
